# Covid-19 vaccine effectiveness during Omicron BA.2 pandemic in Shanghai: A cross-sectional study based on EMR

**DOI:** 10.1097/MD.0000000000031763

**Published:** 2022-11-11

**Authors:** Site Xu, Mu Sun

**Affiliations:** a Ruijin Hospital, Shanghai Jiaotong University School of Medicine, Shanghai, China.

**Keywords:** covid-19, EMR, Omicron BA.2, vaccine effectiveness

## Abstract

Large-scale vaccination against the spread and mutation of COVID-19 is being implemented in many countries. We aimed to assess the effectiveness of certain vaccines (87.35% inactivated), mainly Sinovac – CoronaVac and Sinopharm (Beijing) – BBIBP-CorV, during the Omicron BA.2 pandemic by cross-sectional study. The study was conducted in a cabin hospital of Shanghai, China. A total of 1194 Covid-19 patients infected with Omicron BA.2 were enrolled and epidemiological survey information was collected from the subjects through electronic medical records and questionnaires, from March 23th to April 1st in 2022. Vaccine effectiveness was reflected by the occurrence of multi-dimensional symptoms while adjusting for confounding variables. In the unstandardized vaccinated group, the Covid-19 vaccine effectiveness of Omicron BA.2 in the male group was higher than in the female group (*P* = .0171). In the standardized vaccinated group, vaccine effectiveness in [20, 40) age group was higher than in other age groups (*P* = .0002). Adjusting for gender and age, Covid-19 vaccine effectiveness of Omicron BA.2 at the specific level was 87.42% (95% confidence interval [CI], 72.35–94.28, *P* < .0001), and 62.65% (95% CI, 1.47–85.84, *P* = .047) in the unstandardized vaccinated and the standardized vaccinated group, respectively. Covid-19 vaccine effectiveness of Omicron BA.2 was not apparent at the general level but remained effective for the specific symptom. Further development for the Covid-19 vaccine is necessary.

## 1. Introduction

36 vaccines have been approved for use in response to the COVID-19 pandemic,^[1]^ with over 11.74 billion doses administered globally as of May 18, 2022.^[2]^ Most vaccines have remained effective in preventing severe COVID-19, hospitalization, and death, for all previous variants before Omicron.^[3–5]^ Coronaviruses such as SARS-CoV-2 were relatively stable due to the proofreading mechanism during the replication process, but small errors, including mutations and deletions, still occured,^[6]^ and the replication error correction function of RNA polymerase was limited, making RNA viruses mutated faster. Many variant strains and unknown new variants, which were increasingly different from each other, made it difficult for vaccines to be sufficiently effective,^[7]^ and Omicron has demonstrated the possibility of evading existing vaccines.^[8]^ Given that Omicron had a larger number of mutations than the previous, the potential impact of Omicron on the efficacy of COVID-19 vaccines was not clear.^[9]^

Sinovac – CoronaVac, Sinopharm (Beijing) – BBIBP-CorV, Sinopharm (Wuhan) – Inactivated, CanSino – Ad5-nCOV, Anhui Zhifei – Zifivax, have been authorized for conditional use in China, and were included in WHO’s emergency use listing.^[10–14]^ In China, 3.37 billion doses of COVID-19 vaccines have been administered as of May 19, 2022,^[15]^ the vast majority of which were inactivated vaccines, recombinant subunit vaccines, and adenovirus vaccines. In the study, 87.35% (1043/1194) patients received inactivated vaccines. Among them, Sinovac – CoronaVac and Sinopharm (Beijing) – BBIBP-CorV accounted for 89.55% (934/1043). Evidence of studies from in Chile,^[16]^ Brazil,^[17]^ and China^[18,19]^ showed that the vaccines used in China effectively prevent previous generations of COVID-19, with greater effectiveness against more severe outcomes. However, the efficacy of vaccines against Omicron was unknown, and the confounding factors have not been determined.

To fill this knowledge gap, we aimed to assess the effectiveness of certain vaccines during the Omicron BA.2 pandemic by cross-sectional study.

## 2. Methods

The transmission of Covid-19 was being blocked by centralized isolation in Shanghai. The study was conducted in a cabin hospital for the isolation of patients with Omicron BA.2. 1194 infected people were enrolled. The epidemiological survey information was collected from Named Entity Recognition results of EMR or through questionnaires, from March 23th to April 1st in 2022. The following data were collected: gender, age, height, weight, occupation, city of residence, vaccination times, vaccination type, date of vaccination, date of infection, symptom, body temperature, blood pressure, heart rate, oxygen saturation, respiration, disease history, complication & comorbidity, etc.

If the number of vaccinations reached three times,^[20]^ and the vaccination interval fulfilled the following 3 conditions, the vaccination can be considered to completely reach the vaccination standard: the second dose was more than 4 weeks away from the first dose and within 8 weeks after the first; the third dose was more than 4 weeks away from the first dose and within 6 months after the first; the positive time was within 6 months after the third dose.^[21]^ Since there were too few data that fully met the standard, the study separated standardization statuses as follows: the data that reached two or more conditions were regarded as standardized vaccinated; patients without vaccination were regarded as unvaccinated; the others were regarded as unstandardized vaccinated.

Vaccine effectiveness (VE) was reflected in the occurrence of symptoms. The accordance of positive label included whether there were symptoms (at the general level) and whether each specific symptom has occurred (at the specific level). The data was regarded as positive at the general level once any symptoms occurred, and the data was regarded as positive at the specific level once the specific corresponding symptom occurred. More symptoms were considered more severe at the general level, which also means more ineffective the vaccine was. Specific symptoms included: expectoration, cough, chest pain, chest tightness, palpitations, shortness of breath, dizziness, nausea, vomiting, abdominal pain, diarrhea, abdominal distension, frequent urination, urgency, other (fever, loss of taste, loss of smell, nasal congestion, etc.).

VE was defined by calculating the occurrence rate of symptoms among groups of different standardization statuses and determining the percent reduction in the occurrence rate of the standardized vaccinated group relative to the unvaccinated group. The formula is as follows:^[22]^

Vaccine effectiveness (%) = (1 − odds ratio) × 100

The study focused on the relationship between the standardization status of vaccination and the positive result (i.e., positive label).

### 2.1. Statistical analysis

At the general and the specific level, significant differences were calculated with different positive labels. Chi-square was used for the qualitative, and *t* test or ANOVA was used for the quantitative. Based on preliminary results, logistic regression was used for inclusion factors. A 95% confidence interval (CI) was calculated for each odds ratio and VE. All statistical analyses were performed using Python. *P* < .05 were considered significant.

## 3. Results

### 3.1. Demographic characteristics of enrolled participants with different statuses of standardization

Of the 1194 enrolled participants, 200 (16.75%) were standardized vaccinated, 893 (74.49%) 998 were unstandardized vaccinated and 101 (8.46%) were unvaccinated. Demographic characteristics of enrolled participants, according to the standardization status of vaccination, were summarized in Table 1.

**Table 1:** Demographic characteristics of enrolled participants, according to standardization status of vaccination.

	Unvaccinated (N = 101, 8.46%)	Unstandardized vaccinated (N = 893, 74.79%)	Standardized vaccinated (N = 200, 16.75%)	*P* value
Gender				
Male	64 (8.82%)	545 (75.07%)	117 (16.12%)	.6897^*^
Female	37 (7.91%)	348 (74.36%)	83 (17.74%)	
Age, yr	43 (22)	42 (20)	46.5 (19.25)	.0049^†^
Age				
<20	6 (18.75%)	26 (81.25%)	0 (0%)	.0003^*^
[20,40)	36 (7.33%)	378 (76.99%)	77 (15.68%)	
[40,60)	47 (7.63%)	456 (74.03%)	113 (18.34%)	
>60	12 (21.81%)	33 (60.00%)	10 (18.18%)	
BMI				
Underweight (<18.5)	8 (19.05%)	30 (71.43%)	4 (9.52%)	.499^*^
Fit [18.5, 24)	50 (9.16%)	414 (75.82%)	82 (15.02%)	
Overweight [24, 28)	31 (7.26%)	317 (74.24%)	79 (18.50%)	
Obesity (≥28)	12 (6.70%)	132 (73.74%)	35 (19.55%)	
Vaccination type				
Sinovac	/	375 (85.23%)	65 (14.77%)	<.0001^*^
Sinopharm (Beijing)	/	420 (85.02%)	74 (14.98%)	
Sinopharm (Wuhan)	/	33 (38.37%)	53 (61.63%)	
CanSino	/	27 (100%)	0 (0%)	
Anhui Zhifei	/	14 (%)	1 (%)	
Mixed	/	22 (81.48%)	5 (18.52%)	
Unknown	/	2 (50%)	2 (50%)	
CC				
0-CC	69 (6.96%)	754 (76.01%)	169 (17.04%)	<.0001^*^
1-CC	24 (13.71%)	125 (71.43%)	26 (14.86%)	
2-CC	6 (17.14%)	14 (40%)	15 (42.86%)	
3-CC	2 (100%)	0 (0%)	0 (0%)	

Data are presented as number (%), mean ± standard deviation for normal distribution, or median (interquartile range) for abnormal distribution.

* *P* value from χ^2^ statistic.

† *P* value from ANOVA statistic.

### 3.2. Screening for confounding variables affecting positive results

Of the 1194 enrolled participants, 695 (58.21%) were labeled as positive at the general level and 499 (41.79) were labeled as negative. Positive results at the general level, according to demographic characteristics, were summarized in Table 2. The positive proportion of the female group was significantly higher than that of the male group at the general level (*P* = .0061). The number of positive in different age groups also had significant differences (*P* = .0013).

**Table 2:** Positive results in the general level, according to demographic characteristics of enrolled participants.

	General positive (N = 694, 58.12%)	General negative (N = 500, 41.88%)	*P* value
Gender			
Male	391 (53.86%)	335 (46.14%)	.0061^*^
Female	304 (64.96%)	164 (35.04%)	
Age, yr	41 (19)	45 (19)	.0001^†^
Age			
<20	14 (43.75%)	18 (56.25%)	.0013^*^
[20,40)	178 (36.25%)	313 (63.75%)	
[40,60)	275 (44.64%)	341 (55.36%)	
>60	33 (50.77%)	32 (49.23%)	
BMI			
Underweight (<18.5)	21 (50%)	21 (50%)	.4018^*^
Fit [18.5, 24)	330 (60.44%)	216 (39.56%)	
Overweight [24, 28)	241 (56.44%)	186 (43.56%)	
Obesity (≥28)	102 (56.98%)	77 (43.02%)	
Vaccination type			
Sinovac	250 (56.82%)	190 (43.18%)	.3917^*^
Sinopharm (Beijing)	299 (60.53%)	195 (39.47%)	
Sinopharm (Wuhan)	55 (63.95%)	31 (36.05)	
CanSino	13 (48.15%)	14 (51.85%)	
Anhui Zhifei	8 (53.33%)	7 (46.67%)	
Mixed	18 (66.67%)	9 (33.33%)	
Unknown	1 (25%)	3 (75%)	
Times			
0-dose	50 (49.5%)	51 (50.5%)	.1244^*^
1-dose	20 (50%)	20 (50%)	
2-dose	227 (61.35%)	143 (38.65%)	
3-dose	397 (58.13%)	286 (41.87%)	
Standardization status			
Unvaccinated	50 (49.5%)	51 (50.5%)	.1678^*^
Unstandardized vaccinated	529 (59.24%)	364 (40.76%)	
Standardized vaccinated	115 (57.5%)	85 (42.5%)	
CC			
0-CC	569 (57.36%)	423 (42.64%)	.5829^*^
1-CC	110 (62.86%)	65 (37.14%)	
2-CC	14 (56%)	11 44(%)	
3-CC	1 (50%)	1 (50%)	

Data are presented as number (%), mean ± standard deviation for normal distribution, or median (interquartile range) for abnormal distribution.

* *P* value from χ^2^ statistic.

† *P* value from *t* test statistic.

Positive results in the specific level, according to the standardization status of vaccination, were summarized in Table 3. Positive results of chest pain in different standardization statuses had significant differences (*P* = .0249).

**Table 3:** Positive results in the specific level, according to standardization status of vaccination.

	Unvaccinated (N = 101, 8.46%)	Unstandardized vaccinated (N = 893, 74.79%)	Standardized vaccinated (N = 200, 16.75%)	*P* value
Expectoration				
Positive	37	412	94	.1712
Negative	64	481	106	
Cough				
Positive	12	158	38	.2798
Negative	89	735	162	
Chest pain				
Positive	3	11	7	.0249
Negative	98	882	193	
Chest tightness				
Positive	4	22	7	.5377
Negative	97	871	193	
Palpitations				
Positive	0	3	0	.6024
Negative	101	890	200	
Shortness of breath				
Positive	0	5	1	.7524
Negative	101	888	199	
Dizziness				
Positive	8	68	8	.183
Negative	93	825	192	
Nausea				
Positive	1	15	1	.4131
Negative	100	878	199	
Vomiting				
Positive	1	6	0	.4551
Negative	100	887	200	
Abdominal pain				
Positive	1	5	0	.4609
Negative	100	888	200	
Diarrhea				
Positive	2	22	8	.4304
Negative	99	871	192	
Abdominal distension				
Positive	1	3	0	.3734
Negative	100	890	200	
Frequent urination				
Positive	0	3	0	.6024
Negative	101	890	200	
Urgency				
Positive	0	2	0	.7135
Negative	101	891	200	
Others				
Positive	10	88	13	.3282
Negative	91	805	187	

*P* value from χ^2^ statistic.

Based on the above, in addition to the standardization status, the study included gender and age as confounding variables. Since the number of positive between different standardization statuses did not differ significantly at the general level (*P* = .2345), the study used delamination observation at the general level and used logistic regression incorporating confounding variables for chest pain at the specific level.

### 3.3. Delamination observation in the general level

Table 4 showed the positive results in the general level between different genders in different standardization statuses. In the unstandardized vaccinated group, the positive proportion of the female group was significantly higher than that of the male group (*P* = .0171). Table 5 showed the positive results in the general level between different age groups in different standardization statuses. In the standardized vaccinated group, the positive proportion of [20, 40) age group was significantly higher than that of other groups (*P* = .0002).

**Table 4:** Positive results in the general level, according to gender stratification in different standardization statuses.

	General positive (N = 694, 58.12%)	General negative (N = 500, 41.88%)	*P* value
Standardized vaccinated			
Gender			
Male	61	56	.506
Female	54	29	
Unstandardized vaccinated			
Gender			
Male	298	247	.0171
Female	231	117	
Unvaccinated			
Gender			
Male	31	33	.9397
Female	19	18	

*P* value from χ^2^ statistic.

**Table 5:** Positive results in the general level, according to age stratification in different standardization statuses.

	General positive (N = 694, 58.12%)	General negative (N = 500, 41.88%)	*P* value
Standardized vaccinated			
Age			
<20	0	0	.0002
[20,40)	55	22	
[40,60)	59	54	
>60	1	9	
Unstandardized vaccinated			
Age			
<20	15	11	.1944
[20,40)	238	140	
[40,60)	260	196	
>60	16	17	
Unvaccinated			
Age			
<20	3	3	.8111
[20,40)	20	16	
[40,60)	22	25	
>60	5	7	

*P* value from χ^2^ statistic.

### 3.4. Severity in the general level

**Figure 1.**
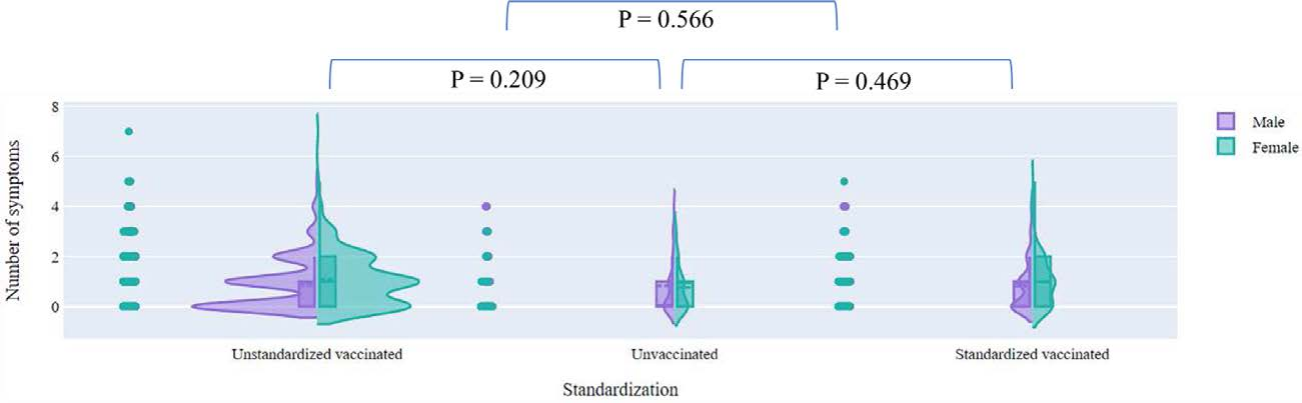
The number of symptoms, according to standardization status of vaccination. Figure 1 showed the number of symptoms in different standardization statuses. No significant difference was shown between different standardization statuses when the severity was reflected by the number of symptoms.

**Figure 2.**
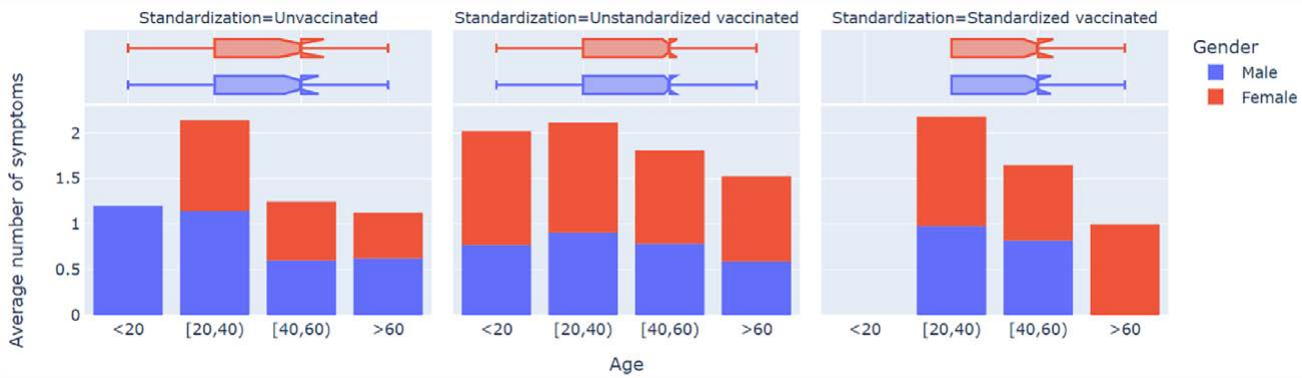
The average number of symptoms, according to standardization status of vaccination. Figure 2 showed the average number of symptoms in different standardization statuses incorporating confounding variables. Variable “age” was distinguished by grouping, and variable “gender” was distinguished by color. Patients in age group [20, 40) have the highest average.

**Figure 3.**
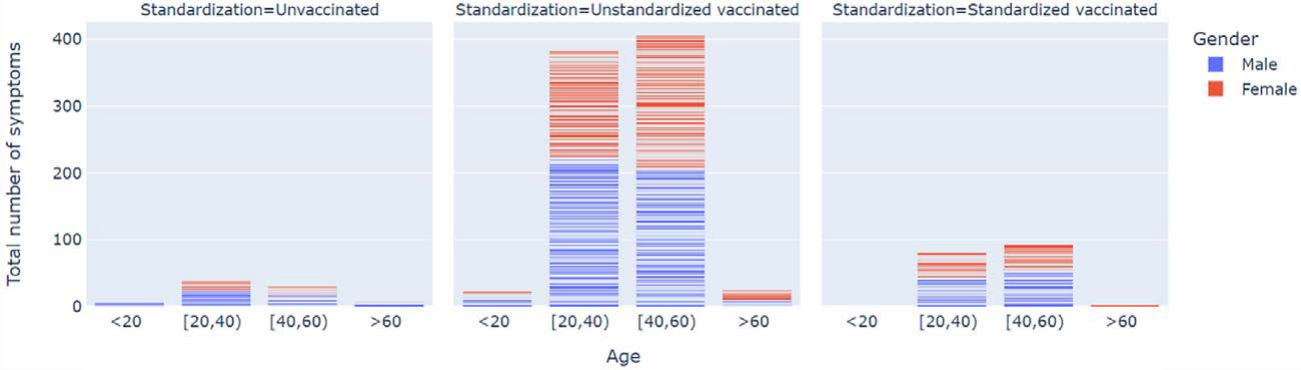
The total number of symptoms, according to standardization status of vaccination. Figure 3 showed the total number of symptoms in different standardization statuses incorporating confounding variables. Variable “age” was distinguished by grouping, and variable “gender” was distinguished by color.

In the study, more symptoms were considered more severe at the general level. Figures 1–3 showed the number of symptoms in different standardization statuses incorporating confounding variables. No significant difference was shown between different standardization statuses when the severity was reflected by the number of symptoms.

### 3.5. Vaccine effectiveness in the specific level

The crude effectiveness against chest pain of unstandardized vaccination was 98.64% (95% CI: 97.59–99.23; *P* < .0001) (Table 6), whereas that of standardization vaccination was as 96.37% (95% CI: 92.29–98.29; *P* < .0001). Statistically significant differences were unchanged after adjusting for gender and age, except for increment VE. The adjusted VEs of unstandardized and standardized vaccination were 87.42% (95% CI: 72.35–94.28; *P* < .0001) and 62.65% (95% CI: 1.47–85.84; *P* = .047), respectively.

**Table 6:** Vaccine effectiveness against chest pain according to standardization status of vaccination.

	No. of positive (N = 694)	No. of negative (N = 500)	Positive rate (%)	Crude VE (95% CI)^*^	*P* value	Adjusted VE (95% CI) ^†^	*P* value
Unvaccinated	50	51	49.5	Reference	/	Reference	/
Unstandardized vaccinated	529	364	59.2	98.64 (97.59–99.23)	<.0001	87.42 (72.35–94.28)	<.0001
Standardized vaccinated	115	85	57.5	96.37 (92.29–98.29)	<.0001	62.65 (1.47–85.84)	.047
Increment VE (standardized vs unstandardized)	/	/	/	1.36 (0.08–2.41)	<.0001	15.61 (7.58–32.15)	.351

/ = no value for category available, VE = vaccine effectiveness.

* Result of the unadjusted conditional logistic regression model.

† Result of the exact conditional logistic regression, adjusted for gender.

## 4. Discussion

Many recent studies have shown that Covid-19 vaccines can still be effective against previous generations of the virus. However, they also raised concerns about the variation of Omicron.^[23]^ An initial aim of this project was to determine whether existing vaccines can still be effective against Omicron. One interesting finding was that gender and age were more influential factors for positive results than the standardization status of vaccination at the general level. One unexpected result was that “standardized vaccinated” and “unstandardized vaccinated” groups did not differ significantly in positive results at the specific level. Importantly, the Covid-19 vaccine effectiveness of Omicron was not obvious at the general level, but remained effective for the specific symptom. The study confirmed that certain vaccines remained the effectiveness against Omicron in some aspects. In contrast to the findings of vaccination standards, the study did not come up with valid confirmation results. Two possible explanations for these might be that VE was already insufficient or existing vaccination standards were unreasonable. However, these results needed to be interpreted with caution because no experiments have been done on aspects like seroconversion rates of neutralizing antibodies, geometric mean titers, etc.

In most of the previous VE studies, they judged effectiveness from the perspectives of hospitalization days and death. However, for the patients affected with Omicron BA.2 who were mostly mild or asymptomatic, the judgment method in this study was more reasonable. Contrary to our expectations, we found that gender and age were more influential factors for positive results than the standardization status of vaccination at the general level. Meanwhile, we did not find any significant differences between “standardized vaccinated” and “unstandardized vaccinated” patients in positive results at the specific level. The cause of the discrepancy was unknown but could be due to the scarcity of data with patients who completely met vaccination standards; the imperfection of the VE evaluation perspective, etc., which were also limitations of the study. In addition, the limitations of the study also include the lack of representativeness due to insufficient data and single data source; insufficient variables due to the scope and data incompleteness of the epidemiological survey. Although the study was not yet perfect, we believe that the results can still be sufficient to conclude at a practical level.

The present results were significant in at least two major respects, develop new specific vaccines and improve vaccination standards. These findings suggested that existing vaccination strategies that were advanced on a large scale can be supplemented as appropriate. Overall, further studies were warranted to examine the VE and the necessity of an Omicron BA.2-specific vaccine.

## 5. Conclusions

Although the Covid-19 VE of Omicron BA.2 showed statistical significance under certain circumstances or in the specific symptom, it did not show significance in other cases. In conclusion, the Covid-19 vaccine effectiveness of Omicron BA.2 was not obvious at the general level but remained effective for the specific symptom. Thus, further research and development for the Covid-19 vaccine and vaccination standards are necessary.

## Acknowledgments

We gratefully acknowledge each medical worker in the cabin hospital for their help in data collection.

## Author contributions

All authors contributed to designing the study. XS and SM were responsible for the investigation and consultation. XS and XG were responsible for data collection and analysis. XS was responsible for writing the manuscript. The corresponding author SM and XG attest that all listed authors meet authorship criteria. No other individuals meeting the criteria have been omitted. Sun is the guarantor. All authors have read and approved the final manuscript.

**Conceptualization:** Mu Sun.

**Data curation:** Site Xu.

**Formal analysis:** Site Xu.

**Investigation:** Site Xu.

**Methodology:** Site Xu.

**Project administration:** Site Xu.

**Resources:** Site Xu.

**Software:** Site Xu.

**Supervision:** Site Xu.

**Validation:** Site Xu.

**Visualization:** Site Xu.

**Writing – original draft:** Site Xu.

**Writing – review & editing:** Mu Sun, Site Xu.
